# Application of Mesoporous Silica Nanoparticles in Cancer Therapy and Delivery of Repurposed Anthelmintics for Cancer Therapy

**DOI:** 10.3390/pharmaceutics14081579

**Published:** 2022-07-29

**Authors:** Maedeh Koohi Moftakhari Esfahani, Seyed Ebrahim Alavi, Peter J. Cabot, Nazrul Islam, Emad L. Izake

**Affiliations:** 1 School of Chemistry and Physics, Faculty of Science, Queensland University of Technology (QUT), 2 George Street, Brisbane, QLD 4000, Australia; maedeh.koohi@hdr.qut.edu.au; 2 Centre for Materials Science, Queensland University of Technology (QUT), 2 George Street, Brisbane, QLD 4000, Australia; 3 School of Medicine and Dentistry, Griffith University, Gold Coast, QLD 4215, Australia; ebrahim.alavi@griffithuni.edu.au; 4 School of Pharmacy, The University of Queensland, Woolloongabba, QLD 4102, Australia; pcabot@pharmacy.uq.edu.au; 5 School of Clinical Sciences, Faculty of Health, Queensland University of Technology, 2 George Street, Brisbane, QLD 4000, Australia; nazrul.islam@qut.edu.au; 6 Centre for Immunology and Infection Control (CIIC), Queensland University of Technology, Brisbane, QLD 4000, Australia

**Keywords:** mesoporous silica nanoparticle, cancer, drug delivery, anthelmintic, nanomaterial, nanotechnology

## Abstract

This review focuses on the biomedical application of mesoporous silica nanoparticles (MSNs), mainly focusing on the therapeutic application of MSNs for cancer treatment and specifically on overcoming the challenges of currently available anthelmintics (e.g., low water solubility) as repurposed drugs for cancer treatment. MSNs, due to their promising features, such as tunable pore size and volume, ability to control the drug release, and ability to convert the crystalline state of drugs to an amorphous state, are appropriate carriers for drug delivery with the improved solubility of hydrophobic drugs. The biomedical applications of MSNs can be further improved by the development of MSN-based multimodal anticancer therapeutics (e.g., photosensitizer-, photothermal-, and chemotherapeutics-modified MSNs) and chemical modifications, such as poly ethyleneglycol (PEG)ylation. In this review, various applications of MSNs (photodynamic and sonodynamic therapies, chemotherapy, radiation therapy, gene therapy, immunotherapy) and, in particular, as the carrier of anthelmintics for cancer therapy have been discussed. Additionally, the issues related to the safety of these nanoparticles have been deeply discussed. According to the findings of this literature review, the applications of MSN nanosystems for cancer therapy are a promising approach to improving the efficacy of the diagnostic and chemotherapeutic agents. Moreover, the MSN systems seem to be an efficient strategy to further help to decrease treatment costs by reducing the drug dose.

## 1. Introduction

**Figure 1:**
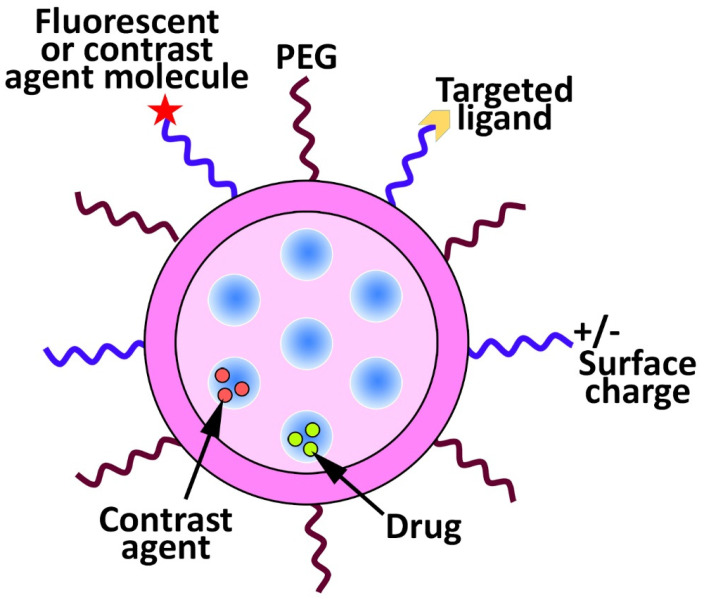
Surface multi-functionalization of mesoporous silica nanoparticles (MSNs) to produce a targeting and stimuli-responsive delivery nanosystem. For this purpose, various types of functionalities, such as polyethyleneglycol (PEG), fluorescent or contrast agents, and targeting ligands (e.g., protein, peptide, and antibody) can be attached to the surface of the nanoparticles. Additionally, the surface charge of the nanoparticles can be modulated, and the nanoparticles can be loaded with various types of drugs and/or contrast agents.

In recent years, significant progress in the biomedical applications of nanotechnology has occurred. More recently, researchers have focused on developing drug delivery systems from nanostructured materials to improve the therapeutic effects of drugs and reduce their side effects [1,2,3]. Liposomes [4], micelles [5], polymeric [6], and silica-based nanoparticles [7] have been investigated to improve drug delivery [8,9]. Mesoporous silica nanoparticles (MSNs) have received considerable attention in biomedicine as carriers for poorly water-soluble drugs in cancer therapy [10]. MSNs have also been used for catalysis [11], magnetism [12], sensors [13], and optical materials [14]. Vallet-Regi et al. [15] were the first who seriously considered MSNs as a controlled drug delivery system [10]. Since then, MSNs have been considered an interesting topic in nanomedicine [16] and have been used to develop more advanced drug delivery systems, namely, theranostics. Theranostics is used for both diagnostic and therapeutic purposes and consists of a therapeutic agent and an optical or magnetic tracer (Figure 1) [10].

**Figure 2:**
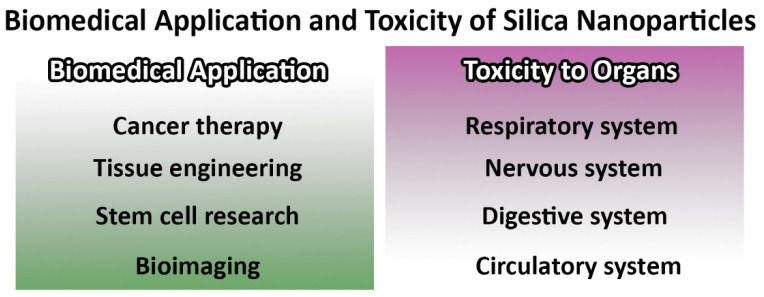
Biomedical applications and toxicity of mesoporous silica nanoparticles (MSNs).

For example, Prasad et al. [17] synthesized folic acid (FA)-modified carbon quantum dot capped (CQD), doxorubicin (DOX)-loaded MSNs (FA-CQD-DOX-MSNs) and evaluated their efficacy for the bioimaging of Hela cancer cells and controlled delivery of DOX into the cells. The results demonstrated that the resulting formulation could prevent premature drug release and excellent Hela cancer cell bioimaging. Kempen et al. [18], in another study, synthesized insulin-like growth factor (IGF)-loaded MSNs, conjugated with fluorescein and gadolinium chloride to develop theranostic MSNs. IGF was loaded into the nanoparticles to enhance cell survival, while ultrasound and magnetic resonance imaging (MRI) signals were used to guide site-specific implantation of the nanoparticles into the murine heart. The in vitro results of this study demonstrated that the labeled human mesenchymal stem cells with these nanoparticles had detection limits of nearly 9000 cells with no cytotoxicity at the required concentration of these nanoparticles (250 µg/mL) for labeling. Additionally, the human mesenchymal stem cells labeled with IGF-loaded MSNs, compared to unlabeled cells, in serum-free culture conditions, could increase cell survival by up to 40% (*p* < 0.05). Overall, the results of this study demonstrated that MSNs were efficient as theranostic agents for the delivery of both the drug and diagnostic agent. MSNs are uniform pore size structures and, due to the large internal surface area, they can decrease drug precipitation and can be administered via various routes, such as oral and intravenous (IV) [7,10,19]. The mesoporous structure and high specific surface area cause MSNs to be loaded with high-capacity drug molecules for both oral and local delivery. The ability of MSNs to release the loaded drug, such as cytochrome c [20] and myricetin [21], under physiological conditions might be even more important [22,23]. MSNs are able to (i) deliver drugs site-specifically [24,25] and (ii) decrease side effects, leading to significant progress in research on their applicability for biomedical use [26]. MSNs are able to transform the crystalline structure of the hydrophobic drugs into an amorphous state, leading to an improvement in the water solubility of the hydrophobic drugs. Additionally, these nanoparticles are able (i) to be modified with various functional groups that can improve their interaction with the loaded drug and, as a result, the profile of drug release, (ii) to be modified with gatekeeper molecules, such as gold nanoparticles to prevent the premature drug release [27], and (iii) to be loaded with various co-therapeutic agents, such as photosensitizer and chemotherapeutics, to develop single and multifunctional anticancer agents [27,28]. This literature review focuses on the applications of MSNs for cancer therapy, in particular on the delivery of anthelmintics as hydrophobic and repurposed drugs for cancer therapy. In addition, the safety issues of MSNs will be discussed in detail (Figure 2).

## 2. Biomedical Applications of Mesoporous Silica Nanoparticles

MSNs have received increasing attention as an ideal candidate for therapeutic uses [27,29,30,31] owing to their promising features such as well-defined pore structure and size, particle morphology, large specific surface area, and framework composition [5]. Moreover, MSNs are exceptionally biocompatible and biodegradable. They are considered as “Generally Recognized as Safe” (GRAS) by the United States Food and Drug Administration (USFDA). Complete degradation of MSNs can occur in one month in simulated body fluid, in the cells, and in the body [27]. Water molecules cause hydrolysis and dissolution of the silica framework that, along with apparent ionic corrosion, participate in mesoporous silica degradation. During this process, the superficial silanol groups of the silica energetically contribute to the formation of a hydrogen bond with water, and the ionic exchange occurring between the superficial mesoporous silica and the degradation medium enhances the process of degradation [32]. MSNs have resistance to various stresses such as pH, mechanical, and thermal stresses [27]. Based on the pore system and surface modification, MSNs can be modified with different gatekeeper molecules, such as inorganic, organic, self-gated drug molecules, and biological membranes, to develop formulations with on-command drug release that can selectively deliver antitumor drugs [33]. For example, Liang et al. [34] developed a d-α-tocopheryl polyethylene glycol 1000 succinate (TPGS) functionalized-MSNs system for the delivery of an Oxaliplatin prodrug (Oxa(IV)). Oxa(IV) was used to reduce the side effects of Oxaliplatin and loaded into the nanoparticles via a covalent bond. TPGS was used as a P-glycoprotein (P-gp) inhibitor. When the resulting formulation (Oxa(IV)-MSNs-TPGS) was endocytosed into cancer cells, Oxa(IV) prodrug was released and reduced to Oxaliplatin by biological stimuli, and the released TPGS inhibited the P-gp activity. In this formulation, the drug itself controlled its release without the need for an additional gating agent. The toxicity and efficacy of Oxa(IV) and Oxa(IV)-MSNs-TPGS were evaluated against human lung adenocarcinoma A549 cells and Oxaliplatin drug-resistant A549 cells (A549/L-OHP) in vitro. The results demonstrated that Oxa(IV), compared to Oxaliplatin, caused significantly lower toxicity by ~7–9-fold in A549 cells. Additionally, the toxicity of Oxa(IV) in A549/L-OHP, compared to that of A549 cells, decreased by ~85%. However, Oxa(IV)-MSNs-TPGS, compared to Oxaliplatin, was more efficient in exerting cytotoxicity in A549/L-OHP cells [34]. Zeng et al. [35] synthesized drug-self-gated MSNs using a pH-sensitive dynamic benzoic–imine moiety. The nanoformulation was loaded with DOX and could demonstrate an appropriate profile of drug release at tumor tissues/cells. The resulting formulation (M-CHO-DOX@DOX) could enhance the anticancer effects of the drug in a pH-dependent manner against human cervical cancer HeLa cells, in which the cytotoxicity of the formulation increased at pH 6.8, compared to that of pH 7.4 (e.g., ~10.4% increase at the drug concentration of 0.25 µg/mL). To further improve the efficacy of the formulation, M-CHO-DOX@DOX was PEGylated, which could improve the antitumor of DOX in a HeLa cell xenograft tumor model in vivo (tumor weight of ~0.71, ~0.28, ~0.13, and ~0.07 g in saline, free DOX, M-CHO-DOX@DOX, and M-CHO-DOX@DOX-PEG groups, respectively) [35]. PEG is a synthetic nontoxic polymer and is able to improve the aqueous solubility of conjugates [36,37,38]. PEG also could improve the in vivo half-life and profile of drug release from nanoparticles [39,40]. Cheng et al. [41] synthesized folic acid (FA)-decorated, DOX-gated, mesoporous silica nanocore containing P-gp small interfering RNA (siRNA) and a polydopamine (PDA) outer layer as a multifunctional nanoplatform, which integrates chemo-(DOX), gene-(P-gp siRNA), and photothermal (PDA layer) substances in one system. The in vitro results demonstrated that the formulation could release DOX in a pH- and thermal-dependent manner. Additionally, P-gp was released from the formulation in a pH-dependent manner owing to the pH-cleavable DOX gatekeeper activity [41]. In addition, the conjugation of PDA and FA as near-infrared light-responsive and selective cell-targeting agents, respectively, into the nanoparticles caused the nanoparticles to be excellent photothermal and selective cell-targeting agents, respectively; thus, in both in vitro and in vivo antitumor experiments, the nanoparticles could enhance antitumor efficacy, in which the resulting formulation (M-R@D-PDA-PEG-FA-D), compared to DOX and DOX + P-gp siRNA, caused ~50- and ~122-fold increase in the cytotoxicity against the drug-resistant human breast cancer MCF-7/ADR cells after 24 h and 48 h incubation, respectively. Additionally, (M-R@D-PDA-PEG-FA-D), compared to DOX + P-gp siRNA, caused a ~19-fold increase in anticancer effects in a xenograft mouse model of breast cancer. Li et al. [42] developed a novel nanomedicine using mannose-doping DOX-loading MSNs (MSN-Man-DOX), coated with polydopamine-Gd3+ (PDA-Gd) metal–phenolic networks and modified by poly (2-Ethyl-2-Oxazoline). The resulting formulation (MSN-Man-DOX@PDA-Gd-PEOz) demonstrated a synergistic effect of chemotherapy and photothermal therapy and worked as a magnetic resonance imaging contrast agent for disease (a xenograft mouse model of lung cancer) monitoring. Various applications of MSNs in nanomedicine have been confirmed, including tissue engineering, wound healing and antibacterial effects [43,44,45], bioimaging [46,47,48], stem cell research [49,50,51], and anticancer/tumor therapy [52,53,54,55,56].

### 2.1. Cancer/Tumor Therapy

Currently, there are various strategies (e.g., chemotherapy, gene therapy, radiation therapy, and surgery) for cancer treatment [57]. The aim of these strategies is to target the cancerous cells, in which the efficient destruction of cancerous cells can occur while normal cells receive the least damage [27]. However, various shortcomings, such as poor solubility, undesirable pharmacokinetics, poor distribution, multidrug resistance (MDR), non-specificity (causing off-target side effects), and instability, cause the failure of these strategies to treat cancer [57]. To overcome these issues, the use of targeted drug delivery systems is a promising approach. However, the efficacy of most of these systems is limited due to premature drug release such as, for example, liposome instability. To address this problem, many efforts have been made to control the release of anticancer drugs to tumors [27,58,59]. Additionally, the low water solubility of hydrophobic anticancer drugs is one of the most severe challenges in anticancer therapy that restrict their IV administration. MSNs have shown promising efficacy in overcoming the insolubility problem of hydrophobic drugs. For example, camptothecin (CPT) and paclitaxel (PTX) have demonstrated exceptional anticancer activity in vitro, but they have shown low anticancer effects in vivo owing to their low water solubility [60,61]. When MSNs are used as a carrier of these drugs, they cause a significant increase in drug solubility due to the tunable pore size and shape of the carrier. The MSN carrier also increases the cytotoxic effects of the drugs by ~86% for CPT against human pancreatic cancer Capan-1 cells and by 4.3-fold for PTX against human liver cancer HepG2 cells [60,61]. Babaei et al. [62] synthesized CPT-loaded polyethylene glycol (PEG)ylated MSNs (PEG@MSNR-CPT) and evaluated their efficacy, compared to the CPT, in the treatment of C26 tumor-bearing mice. The study demonstrated that PEG@MSNR-CPT, compared to CPT, caused a ~19% decrease in the tumor volume. Liu et al. [63] synthesized chondroitin sulfate (ChS)-coated MSNs loaded with PTX and compared the resulting formulation (MSNs-ChS@PTX) to PTX. They showed that the MSNs-ChS@PTX formulation causes a ~24% decrease in tumor volume in an adriamycin-resistant variant of human breast cancer, MCF-7 (i.e., MCF-7/ADR), tumor-bearing mice. MSNs can also be multi-functionalized to control the release of drugs. Bhavsar et al. [64] synthesized DOX-loaded MSNs nanoparticles, functionalized the formulation with cystamine dihydrochloride, and then capped the nanoparticles with chitosan-folate to develop redox and pH-responsive nanoparticles that can actively target breast cancer cells. The drug was released from the particles upon exposure to acidic–redox conditions (cancer environment; pH 5.5, 10 mM reduced glutathione (GSH)). The formulation was found to be safe for IV administration.

MSNs can be surface-modified with PEG to increase their circulation time in vivo and improve the preferential accumulation of drugs into the cancer cells [65]. Additionally, these nanoparticles can be loaded with MDR inhibitors, such as TPGS copolymers, to overcome MDR in cancer cells [65]. Zhao et al. [65] modified the surface of MSNs with TPGS and evaluated their efficacy in overcoming an MDR cancer cell line (DOX-resistant MCF-7/ADR cells). The results demonstrated that DOX@MSNs-TPGS caused a 10-fold increase in cell-killing efficacy when compared to free DOX and DOX@MSNs.

The high stability of MSNs can protect the loaded drug and improve its stability [66,67]. MSNs can also be functionalized with various co-therapeutic agents, such as photosensitizer, photothermal reagents, and chemotherapeutics, to develop single- and multimodal anticancer therapeutics [27,28].

#### 2.1.1. MSNs for Photodynamic (PDT) and Sonodynamic (SDT) Therapies

**Figure 3:**
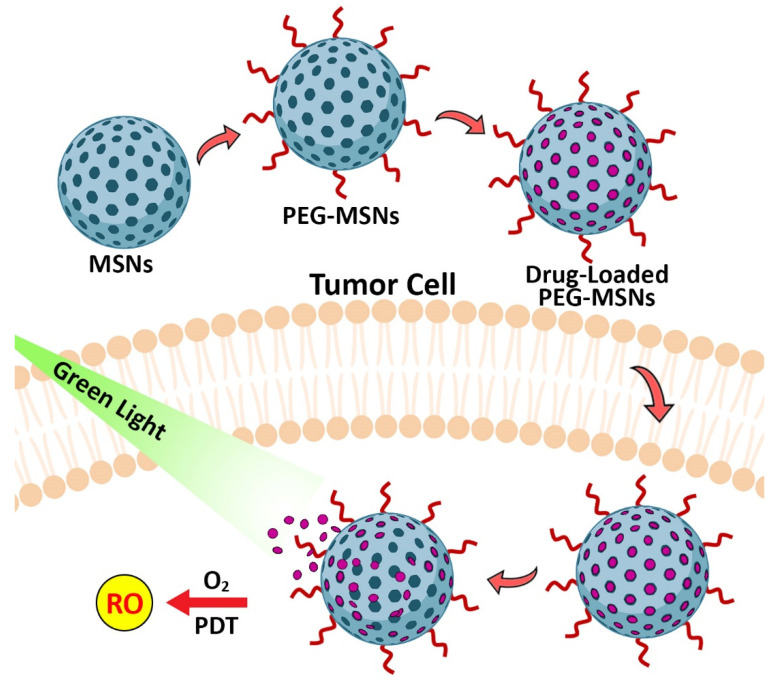
Application of MSNs for photodynamic therapy (PDT) of cancer. The delivery of photosensitizers into tumor cells using PEG-modified MSNs and stimulating the release of photosensitizers by green light irradiation causes the production of reactive oxygen and cancer cell ablation.

Photodynamic therapy (PDT) is a method, in which a photosensitive molecule (i.e., a photosensitizer (PS)) is used in combination with a light source of a specific wavelength [68]. This method is used for the treatment of cancer by utilizing PS as a drug that is activated by light radiation. The activated PS transfers the light energy to molecular oxygen and produces reactive oxygen species (ROS, e.g., single oxygen and free radicals), which kills the nearby cells (Figure 3). PS molecules are nontoxic when they are not activated by light. Therefore, PDT therapy methods that are based on the use of PS as a drug cause fewer side effects, when compared to conventional anticancer therapy methods. However, PDT suffers from some limitations such as: (i) the hydrophobicity of PS molecules that can lead to change in the oxygen quantum fields, (ii) the poor biodistribution of PDT agents in organs results in their accumulation within the skin and cause skin sensitivity [69], (iii) the low tissue penetration of light due to the high tissue absorption of light [70], and (iv) low tumor specificity of PDT agents [69]. MSNs are appropriate drug delivery systems for PDT because they are biocompatible with diverse surface functionalization, and their loading and synthesis can be controlled [27,71,72]. Kuang et al. [73] synthesized PEG-modified MSNs and loaded them with photosensitizer curcumin (Cur). The resulting formulation could increase the anticancer effects of Cur by 4.2-fold against human cervical cancer (Hela) cells.

Sonodynamic therapy (SDT) was developed from PDT as a new, non-invasive treatment method. In this method, low-frequency ultrasound waves are used due to their higher tissue penetration, non-radiative emission and low coefficient of tissue attenuation. SDT has a high capability to eliminate proliferative scars, destroy pathogenic microorganisms and treat solid tumors, leukemia, and atherosclerosis [74,75,76]. Many studies [77,78] have been developed to synthesize more effective MSNs-based sonosensitizers. For this purpose, TiO_2_ MSNs have been chelated with heavy metals, such as Au, Pt, or Ag [79,80,81]. Another commonly used method to produce sufficient ROS for the suppression of tumors’ growth is the development of sono-theragnostic MSNs, which can induce cavitation. Lee et al. [82] produced a nano-photosensitizer of PFH@PEGylated mesoporous silica-titania (P-MSTN) and loaded it with a gas precursor perfluorocarbon. The resulting formulation (PFH@P-MSTN) increased ROS generation and killed tumor cells in response to exposure to ultrasound. PFH@P-MSTN, after systemic administration in tumor-bearing mice, accumulate in tumor site through a passive targeting mechanism [82].

#### 2.1.2. MSNs for Chemotherapy

Chemotherapy is one of the most important therapeutic approaches for cancer treatment. However, the efficacy of traditional chemotherapy is limited due to poor selectivity/solubility of chemotherapeutics, high systemic toxicity, and the development of MDR [27,83]. The development of MDR results from the overexpression of the transmembrane efflux pump P-gp in cancerous cells. MSNs are able to improve the efficacy of chemotherapeutics on their own [27,84,85] and in combination with other modalities of cancer treatments, such as photothermal therapy [86]. MSNs are able to simultaneously deliver anticancer drugs and P-gp inhibitors to tumor tissues [87]. Liu et al. [87] synthesized a nanosystem containing PEG-b-poly(d,l-lactic acid; PEG-b-PDLLA) as a polymer shell responsive to extracellular tumor acidity and a cationic β-cyclodextrin (CD)-polyethyleneimine (PEI; CD-PEI)-modified MSNs. CD-PEI was used as a gatekeeper to release the drug (DOX) in response to the intracellular acidity and glutathione. PEI is able to depolarize mitochondria and causes a disturbance in their respiration and a reduction in their ATP production, thus causing impairment in the P-gp’s ATP-dependent drug-efflux activity. The resulting formulation could cause a significant suppression in the MDR tumor growth with no systemic toxicity. Zhou et al. [88], in another study, developed an injectable matrix based on hollow MSNs (HMSNs) and a thermosensitive PDLLA-PEG-PDLLA hydrogel for the sustained release of Erlotinib (ERT). This injectable matrix (ERT@HMSNs/gel) could transform from a flowing solution to a non-flowing gel structure at room and physiological temperatures, respectively. ERT@HMSNs/gel caused a long-term retention of ERT in vivo and could exert an optimal antitumor effect without systemic toxicity in a mouse non-small cell lung cancer (NSCLC) xenograft model.

MSNs have been widely evaluated for combinational chemotherapy, where they are used as a carrier of phototherapeutics and chemotherapeutics [89,90]. Fang et al. [89] conjugated indocyanine green (ICG) to mesoporous silica-coated gold nanorods (GNR) as a carrier of 5-fluorouracil (5-FU) to develop a multimodal imaging-guided synergistic therapy. ICG was used for fluorescence imaging and PDT. 5-FU was used as a chemotherapeutic agent. The resulting formulation (GNR@SiO2-5-FU-ICG) in the acidic microenvironment of the tumor underwent the protonation of surface silanols, which caused loosening of the electrostatic interaction between 5-FU and the silica shell. Furthermore, the laser-induced heat caused a dissociation in the electrostatic interaction between 5-FU and the silica shell. The release of the 5-FU drug was stimulated by both intracellular acidity and the NIR irradiation. The NIR irradiation induces the production of singlet oxygen and heat that are used for PDT and photothermal therapy, respectively. The GNR@SiO_2_-5-FU-ICG formulation also increases the cytotoxicity of 5-FU by 44% against human melanoma A375 cells. In addition, both 5-FU and GNR@SiO_2_-5-FU-ICG receiver groups were found to decrease the tumor weight in A375 tumor-bearing mice, when compared to the saline receiver group. However, GNR@SiO_2_-5-FU-ICG was more potent by 6-fold in decreasing the tumor weight (1.2, 0.2, and 1.42 g in 5-FU, GNR@SiO_2_-5-FU-ICG, and saline groups, respectively). This study developed a new concept, in which the integration of multiple diagnostic and therapeutic modalities into a single platform increases hopes for a personalized nanomedicine-based strategy [89]. There are various classes of chemotherapeutic drugs, including alkylating agents, antimetabolites, antitumor antibiotics, topoisomerase inhibitors, and tubulin-binding drugs [91]. These chemotherapeutic drugs cause cancer cell death through different mechanisms, such as forming covalent bonds on important molecules (e.g., proteins, DNA and RNA) and binding to tubulin, thereby causing prevention in microtubule formation, which has an important role in mitosis, cell shape, intracellular transport, and axonal function [91].

Anthelmintics are chemotherapeutic agents used for the treatment of infections caused by nematodes [91,92]. Anthelmintics, with various chemical entities, act by changing the metabolism of the parasite (worm) or paralyzing the parasite to enable the host immune system to remove the parasite [93,94]. Some of the anthelmintics can impede the critical oncogenic pathways (e.g., Wnt/b-catenin and nuclear factor kappa-light-chain-enhancer of activated B cells (NF-jB)) [93]. Therefore, anthelmintics may have efficacy in treating cancer [94].

#### 2.1.3. MSN Application in Radiotherapy

Targeted alpha radiation uses radioactive agents, which are selectively delivered to tumors, resulting in minimal damage to peripheral healthy tissues. Clinically various radio nuclides, such as 211At, 213Bi, and 212Pb, and isotopes, such as 225Ac, have been evaluated for targeted alpha therapy [27]. However, the application of these metal complexes may cause cytotoxicity due to their chemical features, such as stability and coordination chemistry. MSNs can be used as a radiotherapy agent due to their targeting ability and bio-compatibility [27,95,96]. For example, Pallares et al. [97] conjugated MSNs into transferrin to increase the nanoparticle concentration at the tumor site and 3,4,3-LI(1,2-HOPO) as a chelator with high selectivity and binding affinity for f-block elements (all metallic elements that have 4f or 5f valence electron subshells), such as actinium (Ac) and plutonium (Pu) [97,98,99]. Transferrin and 3,4,3-LI(1,2-HOPO)-modified nanoparticles were then loaded with two radio isotopes, ^225^Ac and ^238^Pu. The resulting nanoparticles (^225^Ac-HOPO-MSNP and ^238^Pu-HOPO-MSNP) were used to target the human breast cancer BT-549 cells, increase the cytotoxic effects, enhance clearance in vitro, and demonstrate a poor deposition in bones in vivo [97]. Many researchers have attempted to develop combinational tumor therapy using MSNs, such as the combination of radiotherapy with PDT, and X-ray-induced PDT as a derivative of PDT. The new derivative approach, compared to radiotherapy alone, has shown better anticancer effects [100]. Ahmad et al. [101] enhanced the scintillation of CeF_3_ nanoparticles through loading of Tb^3+^ and Gd^3+^ into the nanoparticles (CeF_3_:Gd^3+^, Tb^3+^). The nanoparticles were then coated with MSNs and loaded with Rose Bengal (CGTS-RB). The efficacy of the resulting nanoparticles was then evaluated for CT- and MRI-guided synergistic radiotherapy and PDT using X-ray irradiation. The results demonstrated that X-ray-stimulated synergistic radiotherapy and PDT caused higher tumor regression by ~6-fold in breast cancer-bearing mice when compared to single radiotherapy. Overall, these findings demonstrate the applicability of MSN-based scintillating radio-enhancer nanoparticles for tumor diagnosis and therapy within deep tissues [101].

#### 2.1.4. MSN Application in Immunotherapy

Cancer immunotherapy, as a potent treatment strategy, can inhibit cancer recurrence as it has high antigen-specificity and immune memory [27]. The key point to developing a successful antigen-adaptive immune response is the development of a potent antigen delivery system, which could augment the activation of T cells and dendritic cells. Owing to the high efficiency of MSNs in loading high amounts of cargo, MSNs can be used for the delivery of cancer vaccines containing antigens and adjuvants [102,103]. For instance, Lee et al. [104] developed a core (iron oxide nanoparticles) shell (HMSNs with extra-large mesopores (H-XL-MSNs)) antigen delivery system for augmenting the activation and maturation of dendritic cells. The surface of H-XL-MSNs was coated with poly ethyleneimine (PEI) to modify the surface charge of MSNs, and as a result, to enable the particles for efficient loading and release of antigens. The results demonstrated that the formulation of H-XL-MSNs + PEI + ovalbumin (OVA), compared to soluble PEI + OVA, caused a significant increase (by ~2.9-fold) in the concentration of antigen-specific cytotoxic T cells in vitro. The results also demonstrated that H-XL-MSNs + PEI + OVA, compared to amine-modified H-XL-MSNs + OVA, caused a 5.5% increase in the number of dendritic cells. The in vivo results showed that H-XL-MSNs + PEI + OVA, compared to soluble PEI + OVA, caused a 3.6-fold decrease in the tumor volume in B16-OVA tumor-bearing mice. According to these results, HMSNs with extra-large pores could be considered as an excellent carrier for cancer vaccines.

To further improve the antitumor effects, MSNs could be used as a multifunctional nanoparticle for the combination of immunotherapy with PDT. Xu et al. [105] used biodegradable MSNs (bMSNs) for theragnostic positron emission tomography (PET)-guided PDT and cancer vaccination. The bMSNs were loaded with various multiple neoantigen peptides, CpG oligodeoxynucleotide immune adjuvants, and PS (chlorin e6 (Ce6)). The results of PET imaging in MC38 colon carcinoma-bearing mice demonstrated that bMSN(CpG/Ce6)-neo antigen was preferentially concentrated in the tumor tissue. Furthermore, the results demonstrated that the application of the laser-induced PDT could recruit dendritic cells to the tumor sites and stimulate the responses of neoantigen-specific and tumor-infiltrating cytotoxic T cells. The in vivo results demonstrated that bMSN/vaccine + laser irradiation caused a significant decrease by ~8.9- and ~6.2-fold in the tumor volume of primary and contralateral tumors, respectively.

#### 2.1.5. MSN Application in Gene Therapy

MSNs have been used for the cell-specific delivery of therapeutic nucleic acids to silence or inhibit the expression of certain proteins, which participate in cancer development [27,106]. Nucleic acids are usually unable to cross cell membranes without initial degradation by DNAases or RNAases in the biological milieu. Moreover, they are highly specific toward subcellular compartments. For example, plasmid DNA requires localization at the cell nucleus, while interfering RNA must be delivered into the cell cytoplasm. Thus, MSNs can be used as an excellent carrier for the encapsulation of various genes in their porous structure for endocytosis-mediated cellular uptake [107]. Interestingly, many MSNs used for gene therapy are multifunctional. Du et al. [108] synthesized plasmid DNA (pDNA)-loaded magnetic MSNs (M-MSNs). To protect the loaded pDNA from nuclease degradation, the M-MSNs were modified through grafting to PEI. The PEI-modified pDNA-loaded M-MSNs were then loaded into lipid microbubbles (MBs), and the efficacy of the resulting formulation for in vitro gene transfection and in vivo gene delivery was evaluated. The cytotoxicity results demonstrated that the encapsulation of M-MSNs into MBs caused a significant decrease in the cytotoxic effects of M-MSNs (400 µg/mL) by ~9-fold against human ovarian carcinoma SKOV3 cells. The results of in vitro gene transfection demonstrated that ultrasound (US) + M-MSN@MBs, compared to US, US + MBs, and M-MSNs caused a ~5.2-, ~2-, ~1.9-fold increase in the gene transfection, respectively. Additionally, the results of in vivo gene delivery demonstrated that US + M-MSN@MBs, and US + MBs + pDNA caused ~28.1-, ~5.9-, and ~2.8-fold increase in the gene delivery in ovarian carcinoma-bearing mice when compared to compared to pDNA M-MSNs.

## 3. Anthelmintics for Drug Repurposing

Drug development, compared to finding a new application for an existing approved drug, is probably one of the most challenging and complex efforts in biomedical science. Regardless of the various complex issues that exist behind pharmacological drug designs, enormous difficulties related to clinical, regulatory, intellectual features, and commercial issues must be addressed [109]. Under these challenging conditions, drug development is a slow and uncertain process. To find the alternative for the treatment of diseases, such as cancer, investigators and clinicians turned their attention to the strategy of drug repurposing [109]. Drug repurposing is the process of finding a new application for an existing approved drug. This is an appropriate strategy since pharmacokinetics, pharmacodynamics, and toxicity profiles are well-known for many drugs and, therefore, they have low failure rates due to the side effects [94]. Additionally, the development of a repurposed drug, compared to a new drug, has lower expenditure (50–60%) and needs less time (3–12 versus 10–15 years) [94,110]. Moreover, repurposed drugs, compared to new drug applications, have a higher chance of reaching the market (approximately 10 versus 30%) [111].

According to the literature [112], the repurposing drugs in the oncology (ReDO) project chose 268 drugs as repurposed drugs for cancer treatment. The drugs must be licensed for non-cancer therapy in at least one country throughout the world, and one or more peer-reviewed publications [93,113,114] demonstrated that the drug has specific anticancer effects against one or more malignancies [112].

**Figure 4:**
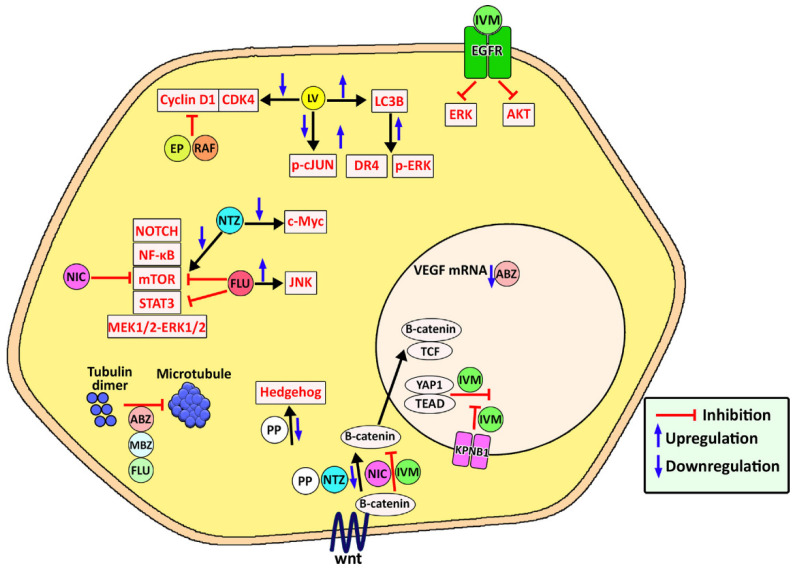
Mode of action used by various anthelmintics to exert their anticancer effects. The anthelmintics work through the inhibition of tubulin polymerization to form microtubule, downregulation of Hedgehog signaling pathway, inhibition and downregulation of wnt/B-catenin pathway, and inhibition of Notch, NF-κB, mTOR, STAT3, and MEK1/2-ERK1/2 signaling pathways; downregulation of c-Myc signaling pathway; upregulation of JNK signaling pathway, inhibition and downregulation of cyclin D1-CDK4 pathway; downregulation of pc-jun pathway; upregulation of LC3B; upregulation of DR4 and p-ERK pathways, and inhibition of ERK and AKT. ABZ, albendazole; IVM, ivermectin; LV, levamisole; MBZ, mebendazole; NIC, niclosamide; FLU, flubendazole; RAF, Rafoxanide; NTZ, nitazoxanide; PP, pyrvinium pamoate; EP, eprinomectin; NF-κB, nuclear factor kappa-light-chain-enhancer of activated B cells; mTOR, mammalian target of rapamycin; STAT3, signal transducer and activator of transcription 3; JNK, c-Jun N-terminal kinase; CDK4, cyclin-dependent kinase 4; LC3B, microtubule-associated protein 1 light chain 3B; DR4, death receptor 4; p-ERK, phosphorylated extracellular signal-regulated protein kinase; AKT, protein kinase B. Reproduced with permission from ref. [94]. Copyright 2021 Elsevier.

Anthelmintics are an important drug class that has demonstrated antitumor activity [94]. These drugs were initially used for parasite treatment in veterinary sciences, and then have been used in humans for the treatment of helminthiasis [94]. These drugs are antiparasite agents, causing a change in the parasite (worm) metabolism or parasite paralysis, thereby resulting in the removal of the parasite by the host immune system [88,89]. They have also demonstrated anticancer activity against various types of cancers, such as leukemia and ovarian cancer. They exert their anticancer effects through various mechanisms, such as down-regulation of the expression of P-gp through the inhibition of the epidermal growth factor receptor (EGFR) [110], disturbing the polymerization of microtubules, apoptosis induction, restricting the cell cycle progression (G2/M), anti-angiogenesis, the blockage of glucose transport, and un-coupling of the mitochondrial oxidative phosphorylation (Figure 4) [115,116]. However, the clinical application of anthelmintics for cancer therapy is limited due to low water solubility and, consequently, low bioavailability [94,117]. MSNs can improve the solubility of hydrophobic drugs by transforming their crystalline structure into an amorphous state. In addition, these nanoparticles can be modified with functional groups, causing an increase in the interaction between the loaded drug and carrier, resulting in slower drug diffusion and sustained drug release [27]. To control drug release, gatekeeper molecules (e.g., gold nanoparticles and bulky proteins) can be utilized to obstruct pore access and, as a result, impede premature cargo delivery. Based on the type of gatekeepers, various internal (e.g., pH, redox state, endogenous enzyme) and external (e.g., heat, sound) stimuli can be used to stimulate the opening of the pore apertures in MSNs and drug release [27].

The excitation of the gatekeepers by stimuli causes them to degrade or bind to the silica surface by readily cleavable bonds without decomposing the MSNs [118]. pH is one of the commonly used stimuli to control drug release into the acidic cancerous cells [119]. In this approach, a pH-responsive gatekeeper is non-covalently bound to the surface of MSNs and inhibits the diffusion of the drug under neutral conditions. The acidic environment of the cancer cells stimulates the covered pores and leads to drug release [27]. The redox state is an internal stimulus that is also used for the stimulation of drug release for cancer treatment [120]. This stimulation is based on the remarkable increase in the intracellular GSH level, compared to the extracellular environment, discerned in most cancers. In this strategy, GSH causes the cleavage of disulfide bonds between the redox-responsive gatekeeper and MSNs, resulting in the opening of the caps and the drug release [27]. Based on chemical structures and pharmacological features, anthelmintics are classified into seven groups: benzimidazoles (e.g., thiabendazole, mebendazole, fenbendazole, oxfendazole, oxibendazole, albendazole, ricobendazole, and triclabendazole), pro-benzimidazoles (e.g., netobimin, febantel, and thiophanate), tetrahydropyrimidines (e.g., pyrantel pamoate, pyrantel tartrate, and morantel), imidazothiazoles (e.g., levamisole), iso-quinolines, salicylanilides (e.g., closantel, niclosamide, and rafoxanide), and macrocyclic lactones (e.g., avermectins such as abamectin and milbemycins or endectocides; ivermectin, moxidectin, doramectin, eprinomectin, selamectin, and milbemycin oxime) [115,121]. The use of MSNs as a carrier of some anthelmintic drugs (Table 1) for the treatment of cancers will be discussed in the following section.

### 3.1. Applications of MSNs for the Delivery of Anthelmintic Drugs for Cancer Therapy

#### 3.1.1. Albendazole

Ghaferi et al. [128] synthesized albendazole (ABZ)-loaded Mobil Composition of Matter No. 41 (MCM-41) and evaluated the efficacy of the resulting formulation, compared to ABZ, against human prostate cancer PC-3 cells in vitro. The MCM-41 nanoparticles were synthesized via a sol–gel process, where the surfactant N-cetyltrimethylammonium bromide (CTAB), tetraethylorthosilicate (TEOS), and NaOH were used as the structure-directing agent, a silica precursor, and a base catalyst, respectively [129]. In this process, the silica precursor was hydrolyzed and condensed to form the mesoporous structure [130]. First, the surfactant (CTAB) molecules with the long chain were arranged as a micelle, containing an inner core consisting of hydrophobic tails. The micelles, with the positive charge, then interacted electrostatically with the negatively charged silicate species and, as a result, tubular silica was formed around the micelles [131]. To remove the surfactant template, calcination was preferred to the solvent extraction method as the solvent extraction method can be performed incompletely, resulting in incomplete removal of the surfactant [132]. The results demonstrated that ABZ-loaded MCM-41 with the size and drug loading efficiency of 293 ± 8.7 nm and 30% were synthesized. Drug loading into the nanoparticles caused an increase in the size and zeta potential of the particles, confirming the successful loading of the drug into the nanoparticles. The morphology of the nanoparticles was evaluated by SEM and TEM. The results demonstrated that the nanoparticles were synthesized as homogenous and spherical particles, which had a smooth surface. Additionally, the results of the morphology evaluation demonstrated that the nanoparticles were formed as uniform porous structures. Nitrogen (N2) sorption analysis was used to evaluate the pore properties of the nanoparticles, and the results demonstrated that both MCM-41 and ABZ-loaded MCM-41 had a typical type IV isotherm, which is a characteristic isotherm of mesoporous materials. In this type of isotherms, layer-by-layer adsorption occurs on a smooth, nonporous surface [133]. In the isotherm, a distinct step at a relative pressure (p/p0) of ∼0.2–0.4 was observed that was regarded as a characteristic feature of MCM-41 mesoporous materials [129]. Additionally, the type of drug loading (chemical or physical) was evaluated using FTIR analysis, and the results demonstrated that ABZ preserved its chemical structure after loading into the nanoparticles, indicating that the drug was loaded physically into the particles [128]. The ABZ-loaded MCM-41 formulation was found to increase the cytotoxicity of the drug by 2.9-fold (half-maximal inhibitory concentration; IC_50_ = 23 and 7.9 µM for ABZ and ABZ-loaded MCM-41 nanoparticles, respectively), resulting in a reduction in the required dose, and consequently, treatment cost. Hernández-Castillo et al. [134] loaded ABZ into hybrid chitosan-coated mesoporous silica nanospheres (SiO_2_-MS-ABZ-CBS) and evaluated its anticancer effects against human cervical cancer CaSki cells. The silica nanoparticles were synthesized using the Stöber method and etched to prepare mesoporous particles with a size range of 350–400 nm. The etched nanoparticles, compared to nonetched nanoparticles, had a higher specific BET area (15 m^2^/g to 150 m^2^/g) and uniform pore size distribution. The results of X-ray powder diffraction demonstrated that the nanoparticles were formed as amorphous structures and a low-intensity peak attributed to ABZ. The thickness of the chitosan coating was 10 to 15 nm. The formulation was found to increase the cytotoxic effects of ABZ by ~3.8-fold against CaSki cells. The formulation was found to increase the cytotoxic effects of ABZ by ~3.8-fold against CaSki cells. Adrover et al. [135] also used Santa Barbara Amorphous (SBA-15) and SBA-16 ordered mesoporous silica materials to improve the dissolution rate of ABZ. The results of TEM analysis demonstrated that both SBA-15 and SBA-16 were greatly porous. SBA-15 demonstrated a mean pore size of ~5 nm and a periodical well-organized hexagonal array, while for SBA-16, a mean pore size of ~6 nm was detected. The amorphous state of the loaded ABZ caused a notable increase in the dissolution rate of ABZ. SBA-15, compared to SBA-16, demonstrated an increase in drug-loading efficiency from 12.8% to 30.3 wt% and its dissolution rate from 0.0171 ± 0.0011 mg/mL to 0.0190 ± 0.0013 mg/mL; thus, it can be used as a promising carrier for improving the ABZ bioavailability.

#### 3.1.2. Thiabendazole

Koohi Moftakhari Esfahani et al. [136] used Mobil Composition of Matter Number 41 (MCM-41) as an MSN drug carrier to improve the anticancer effects of thiabendazole (TBZ). The drug-loaded nanoparticles (TBZ MCM-41, size = 215.9 ± 0.07 nm, drug loading capacity = 19.1%) were synthesized, and the amorphous state of the loaded drug was confirmed by differential scanning calorimetry (DSC). The polydispersity index (PDI) of the nanoparticles was found to be 0.209 ± 0.03, indicating that they were homogeneous and monodisperse. The results of SEM and TEM analysis also confirmed the nanoparticles were spherical and homogeneous. The results of FTIR spectroscopy demonstrated that the chemical bands of TBZ were intact in TBZ MCM-41, demonstrating that TBZ was loaded physically into the nanoparticles. The results of N2 adsorption/desorption analyses demonstrated that TBZ MCM-41 had the typical type IV isotherm, which is a characteristic isotherm of mesoporous material [133]. Additionally, the isotherms for both MCM-41 and TBZ MCM-41 formed H1 hysteresis loops, indicating the formation of well-shaped pores with a hexagonal geometry. TBZ MCM-41 was found to increase the anticancer effects of TBZ by 2.8-fold against human prostate cancer PC-3 cells (IC50: 127.3 and 46 μM for TBZ and TBZ MCM-41, respectively). Additionally, the TBZ MCM-41 nanoparticles increased the production of intracellular reactive oxygen species (ROS) in these cells by 1.2-fold when compared to TBZ alone.

#### 3.1.3. Fenbendazole

Koohi Moftakhari Esfahani et al. [53] synthesized fenbendazole (FBZ)-loaded PEGylated MCM-41 nanoparticles (PEG-MCM-FBZ) and evaluated their cytotoxic effects against PC-3 cells. They functionalized the nanoparticles using a post-synthetic grafting method and (3-Aminopropyl)triethoxysilane (APTES) for the amine modification of the nanoparticles. The particles were functionalized through silylation of the surface-free and germinal silanol groups. The amine modification of the nanoparticles produces different surface charges that can be utilized to control the release and delivery of the loaded drug under different pH conditions [129]. The amine-modified nanoparticles were then PEGylated using D-tocopherol polyethylene glycol succinate (TPGS)-carbonyldiimidazole (CDI). The size, zeta potential and drug loading efficiency of the synthesized nanoparticles (PEG-MCM-FBZ) were 366.3 ± 6.9 nm, 24.7 ± 0.4 mV, and 17.2%, respectively. The results of SEM and TEM analyses demonstrated that the nanoparticles were homogenous and monodisperse with smooth morphology that, compared to crystalline and irregular particles, are more appropriate because nanoparticles with smooth morphology have less potential for tissue irritation. The monodispersity of the drug nanocarriers warrants the nanocarriers to have uniform physical, chemical, and biological features for biomedical applications [137]. PEG-MCM-FBZ was found to release FBZ in a controlled manner in both pH 1.2 and 6.8. In addition, the PEG-MCM-FBZ nanoparticles were found to increase the cytotoxic effects of FBZ against PC-3 cells by 3.8-fold when compared to FBZ. PEG-MCM-FBZ was also found to increase the intracellular production of ROS by 1.3-fold when compared to FBZ. The results demonstrated that PEG-MCM-FBZ was more potent than the non-PEGylated counterpart (MCM-FBZ) by 1.4- and 1.3-fold to increase the cytotoxic effects and ROS production, respectively. These results indicate that the PEGylation of MCM-41 nanoparticles is a promising approach to improving the nanoparticles’ therapeutic properties. PEG-MCM-FBZ caused a lower amount of drug release by 9 and 10% at pH 1.2 and 6.8, respectively, when compared to MCM-FBZ. This caused the drug to release for a longer time at both pH values [53]. Koohi Moftakhari Esfahani et al. [138], in another study, synthesized FBZ-loaded Mobil Composition of Matter No. 48 (MCM-48) and functionalized the particles with succinylated β-lactoglobulin (BLG) to prevent early-burst drug release. The FBZ-MCM-BLG formulation (size = 369 ± 28 nm, zeta potential = 28 ± 0.4 mV, drug loading efficiency = 19%) caused the drug to release in a pH-dependent and controlled manner at the pH values of 1.2 and 6.8. FBZ-MCM-BLG also decrease the early burst drug release by 16 and 14% at pH 1.2 and 6.8, respectively, when compared to FBZ-MCM. These results demonstrate that MCM and MCM-BLG could efficiently control the drug release under acidic gastric (pH 1.2) and neutral intestinal (pH 6.8) conditions. Additionally, FBZ-MCM-BLG could increase the cytotoxic effects of the drug by 5.6-fold against PC-3 cells. The authors demonstrated that MCM-48 nanoparticles could be considered as a promising carrier for the delivery of FBZ, and the functionalization of these nanoparticles with BLG could further improve their properties to be more potent than FBZ-MCM and increase the cytotoxic effects and ROS production in PC-3 cells by 1.8- and 1.2-fold, respectively.

#### 3.1.4. Niclosamide

Pardhi et al. [139] synthesized niclosamide-loaded mesoporous silica (Sylysia-350) carrier to improve the dissolution rate of the drug. The Sylysia-350 carrier increased the dissolution rate of the drug by ~2.7-fold (dissolution rate of 37% vs. 100%), and the maximum enhancement in the dissolution rate was found at a drug: carrier loading ratio of 1:2 (w/w). The niclosamide-loaded carrier also caused an increase in the cytotoxicity of the drug by 2.5- and 4.3-fold against human colon cancer HCT 116 and human lung cancer NCI-H460 cells, respectively (IC_50_: 0.19 ± 0.01 mM in HCT-116 and 0.20 ± 0.02 mM in NCI-H460 for nanoformulation and 0.48 ± 0.02 mM and 0.86 ± 0.07 mM in HCT-116 and NCI-H460 cells, respectively for niclosamide).

#### 3.1.5. Avermectins

Avermectins are a group of naturally occurring drugs produced from fermenting *Streptomyces avermitilis*, which are soil actinomycetes. Avermectins generally act by inhibiting the electrical impulse transmission in the muscle and nerves of invertebrates by amplifying the glutamate effects on the gated chloride channel. The avermectins cause side effects when used indiscriminately. These side effects include respiratory failure, hypotension, and coma [140]. The application of mesoporous silica-based materials as carriers for avermectins is discussed in the following section.

##### Ivermectin

Shen et al. [141] used MSNs and polydopamine (PDA)-modified MSNs (MSN-PDA) for the delivery of ivermectin. They used copper (Cu), iron (Fe), and zinc (Zn) ions to introduce a coordination bridge between vehicle and cargo and produced MSN-PDA-Zn, MSN-PDA-Cu, and MSN-PDA-Fe. The drug loading efficiency values for MSNs, MSN-PDA, MSN-PDA-Zn, MSN-PDA-Cu, and MSN-PDA-Fe were 133, 96, 76, 248, and 243 mg/g, respectively. The results also demonstrated that the incorporation of metal ions into the structure of the formulation caused a decrease in the cumulative release rate. In contrast, due to the acid sensitivity of PDA-modified samples, in acidic pH, compared to alkaline pH, a higher amount of the loaded drug was released from AVM/MSN-PDA (34 versus 23% at pH 4 and 10, respectively). Overall, the results of this study demonstrated that the drug loading efficiency was highly dependent on the bridge effect of the metal ions and the acidic environment promoted the drug release from PDA-modified samples due to the acid sensitivity of these formulations. Li et al. [142] loaded ivermectin into porous hollow silica nanoparticles (PHSNs; 140–180 nm) with shell thicknesses ranging from 5 to 45 nm and a pore diameter of ~ 4–5 nm. The efficacy of the resulting formulations in controlling the drug release was evaluated. The results demonstrated that the drug loading efficiency decreased by increasing the shell thickness where nanoparticles with 5, 15, 30 and 45 nm shell thicknesses achieved drug loading efficiencies of 63.6%, 62.5%, 58.3%, and 55.4%, respectively. Increasing the shell thickness also increased the protective activity of the nanoparticles on the ivermectin degradation and decreased the amount of the drug that is released from the nanoparticles. Liang et al. [143] loaded ivermectin into redox and α-amylase dual stimuli-responsive MSNs that are functionalized with starch. The starch was covalently attached to the nanoparticles to protect the drug against photodegradation and inhibit the premature release of the drug. However, metabolizing the nanoparticles with glutathione and α-amylase caused the coated starch and disulfide bridge structures to be degraded and the drug to be released. The results demonstrated that, in the absence of glutathione, only 8.2% of the loaded drug was released, while increasing the glutathione concentration from 2 to 8 mM caused an increase in drug release from 21.7% to 51.3% over 7 days. Additionally, in the absence of α-amylase, less than 9% of the loaded avermectin was released, while the addition of α-amylase caused an increase in drug release by more than 8-fold after 7 days.

##### Abamectin (Avermectin B1)

Feng et al. [144] used MSNs as a carrier of abamectin. The drug-loaded MSNs with the size of 260 nm, spherical shape, rough surface, uniform particle sizes, typical hollow structure, and drug loading efficiency of 44.8% were produced that could release the loaded drug in a controlled manner. While technical abamectin was completely released after 48 h, only ~71% of the loaded drug was released after 120 h. The drug release from MSNs could be influenced by the temperature, pH of the release medium, the particle size, and the drug loading efficiency; thus, the profile of drug release can be adjusted by modifying these parameters. The stability test results demonstrated that MSNs provided a strong protective effect on the loaded drug, in which unloaded technical abamectin as the control exhibited 59.6% decomposition after 9 days, while less than 30% of the loaded drug was decomposed after 9 days. Wang et al. [145], in another study, synthesized abamectin-loaded porous silica nanoparticles (Abam-PSNs) and evaluated the drug release behaviors and photostability of Abam-PSNs. PSNs with the size of 320 nm, a specific surface area of 318.62 m/g^2^, and drug loading efficiency of 111.0 mg/g were synthesized that could efficiently control the release of the drug, in which PSNs could decrease the amount of drug release from 0.227 mg/h in abamectin solution to 0.013 mg/h in Abam-PSNs at the timepoint of 24 h. The results of the stability test demonstrated that PSNs could significantly protect the drug against photolytic degradation, in which the photolytic percentage of the abamectin was 1.4% and 26%, respectively, for the Abam-PSNs and the pure abamectin after 12 h.

## 4. Toxicity of Mesoporous Silica Nanoparticles

By decreasing the size of the materials, their surface-to-volume ratios and surface reactivity are increased [146]; thus, humans are readily exposed to NPs through breathing, eating, and touching. MSNs are able to distribute to various tissues through the blood circulation system; therefore, their toxicity has been extensively studied in vitro and in vivo [147]. In the following sections, the human system- and organ-specific toxicity of MSNs are discussed.

### 4.1. Toxicity to the Respiratory System

Studies [148,149] have shown that both pure and surface-functionalized MSNs are highly biocompatible in vivo. Although in vitro studies [150,151,152] demonstrated that MSNs, at doses of up to 100 μg/mL, are nontoxic, higher doses could cause cytotoxicity. The excess production of ROS is one of the main mechanisms of MSN toxicity [153]. ROS production occurs via the depolarization of the mitochondrial membrane, the impairment of the electron transport chain, and the activation of nicotinamide adenine dinucleotide phosphate (NADPH) oxidases [154]. Maccuaig et al. [155] synthesized MSNs with the size of 25 nm, coated the particles individually with PEG (PEG-MSNs) and chitosan (C-MSNs), and then evaluated their toxicity in mice. The nanoparticles were injected intravenously, and after chronic and acute injection, their lung toxicity was evaluated. After 4 weeks of administration of the nanoparticles, the minor exacerbation of pre-existing lesions in the lung was observed in the 35KDa PEG-MSN receiver group, while the moderate exacerbation of pre-existing lesions was observed in uncoated MSN and 2KDa PEG-MSN receiver groups. In contrast, minimal changes were observed in the C-MSN group when compared to the control group. According to the results of this study, the administration of C-MSN, as either single or multiple intravenous injections, caused minimal toxicity, while PEG-coated nanoparticles, especially 2 KDa PEG-MSNs, could cause exacerbation of pre-existing vascular conditions [155]. In another study, Hozayen et al. [153] evaluated the pulmonary toxicity of MSNs in Wistar rats. The nanoparticles with the size of ~50 nm were administered intraperitoneally for 4 weeks (25, 50, 100, and 200 mg/kg body weight/day). The lung of rats demonstrated a significant increase in ROS, malondialdehyde (MDA), and nitric oxide (NO) production in a dose-dependent manner, in which the rats receiving a nanoparticle concentration of 200 mg/kg body weight/day, compared to those receiving 25 mg/kg body weight/day, demonstrated a ~2.7-, 1.9-, and 2.3-fold increase in ROS, MDA, and NO production, respectively. Additionally, the nanoparticles caused a significant decrease in the antioxidant defenses in the lung of rats in a dose-dependent manner, in which the concentration of reduced glutathione (GSH), superoxide dismutase (SOD), catalase (CAT), and glutathione peroxidase (GPx) in the lung of rats receiving the nanoparticle concentration of 200 mg/kg body weight/day, compared to those receiving the nanoparticle concentration of 25 mg/kg body weight/day, decreased by ~1.8-, ~1.7-, ~1.8-, and ~3-fold, respectively. Additionally, the results of the histopathological study demonstrated that the lung histopathological lesions such as peribronchiltis were more severe in the rats receiving the nanoparticle concentration of 200 mg/kg body weight/day, compared to those receiving the nanoparticle concentration of 25 mg/kg body weight/day. Overall, according to the results of these studies, it can be suggested that the lung toxicity of MSNs depends on the nanoparticle structure and nanoparticle concentration.

### 4.2. Toxicity to the Nervous System

#### 4.2.1. Brain

Brain tissue mostly consists of neurons and glial cells; thus, the central nervous system (CNS), in particular, is susceptible to being damaged due to ROS-induced oxidative stress [156]. The smaller size of nanoparticles, in combination with the specific proteins of apolipoproteins that contribute to the formation of the protein corona, permit the nanoparticles to pass through the blood–brain barrier [157] and attain the CNS through the nasal epithelium [158]. Zhou et al. [156] synthesized various surface-modified MCM-41 nanoparticles, including MSN-OH (278.5 nm), MSN-NH2 (266.1 nm), MSN-NH2-SH (271.5 nm), and MSN-SH (247.4 nm). MSN-NH2 was also covalently linked to the fluorescent dye Cy5.5 (MSN-Cy) and then covalently attached to transferrin (MSN-Cy-Tf). The neuronal damage and neurotoxicity effects of the nanoparticles were evaluated in vitro and in vivo. The in vitro results demonstrated that various surface-modified MSNs caused a decrease in the viability of rat neuronal PC12 cells in a surface and concentration-dependent manner, in which MSN-OH with the half-maximal inhibitory concentration (IC_50_) of 5.8 μg/mL caused the highest cytotoxicity (IC_50_: 5.8, 26.9, 27.1, and 48.7 μg/mL for MSN-OH, MSN-NH_2_, MSN-NH2-SH, and MSN-SH, respectively). Additionally, to evaluate the molecular mechanism of neuronal damage, the levels of intracellular ROS, intracellular GSH, extracellular lactate dehydrogenase (LDH), and intracellular MDA in the MSN-treated PC12 cells were measured. The results demonstrated that MSNs in a surface- and concentration-dependent manner caused an increase in the intracellular concentration of ROS, in which MSN-OH, MSN-NH_2_, MSN-NH_2_-SH, and MSN-SH at the IC_50_ concentration caused an increase in the intracellular concentration of ROS by 2.5-, 2.5-, 1.4-, and 1.1-fold, compared to the control group. Additionally, the nanoparticles at the IC_50_ concentration caused a decrease in the GSH content, in which MSN-OH-, MSN-NH_2_-, MSN-NH_2_-SH-, and MSN-SH-treated cells could remain as 64.6%, 48.0%, 55.9%, and 33.2% of the intracellular GSH, respectively, compared to the control group. Additionally, the effects of the nanoparticles on the extracellular concentration of LDH were evaluated, and the results demonstrated that there was no significant difference between the nanoparticles in the production of extracellular LDH. Additionally, the effects of the nanoparticles on the intracellular production of MDA were evaluated, and the results demonstrated that all the nanoparticles caused a concentration- and surface-dependent effect on MDA production. All of the nanoparticles at the IC_50_ concentration caused a 4–5-fold increase in the MDA generation, compared to the control group. The in vivo imaging results demonstrated that both MSN-Cy and MSN-Cy-Tf nanoparticles could enter the brain, and the histological observation of the hippocampus confirmed the delivery of MSNs into the CNS that caused neuronal damage in the form of neuronal cell loss, nuclei shrinkage, and neurons disintegration, indicating the in vivo neurotoxicity. In another study, Chauhan et al. [159] studied the effects of MSNs in bone marrow mononuclear cells (BM-MNCs) and human neuroblastoma SH-SY5Y cells and found that the nanoparticles (123 nm) at the low concentration (5–25 µM) were nontoxic towards BM-MNCs, while the concentration of 50–100 µM caused cytotoxicity after 2 h of incubation. Regarding the SH-SY5Y cells, it was found that the MSNs were nontoxic at 1–10 ng/μL after 24 h of incubation, while the nanoparticles were toxic at the concentration of 1–100 µg/μL after 48 and 72 h. According to the results of these studies, the surface chemistry and concentration of MSNs play a critical role in their brain toxicity.

#### 4.2.2. Eye

The eye is a superficial organ, by which the majority of exoteric information (90%) is obtained. The continuous exposure of eyes to potential environmental toxins such as gases and nanomaterials can result in severe adverse reactions in the ocular system [160]. The cornea is the most external physical barrier of the ocular surface [161] and works as the first line of defense against invading foreign materials to the eye surface. Chen et al. [160] demonstrated that Ag+-loaded MSNs and MSNs with the size of 86.3 nm caused toxicity against human corneal epithelial hCEC cells in a concentration- and structure-dependent manner, in which, by increasing the nanoparticles concentration, the cytotoxicity also increased and Ag+-loaded MSNs, compared to MSNs, caused higher toxicity (e.g., by 18.2% at the concentration of 100 µg/mL). Additionally, Ag+-loaded MSNs, compared to MSNs, caused more severe corneal damage and dry eye (by ~15%) in a rat model.

### 4.3. Toxicity to the Digestive System

#### 4.3.1. Liver

The liver is one of the most perfused organs and has a critical role in the metabolic function of the digestive system [147]. The liver toxicity of MSNs has been studied in vitro and in vivo. Zhang et al. [162] evaluated the hepatotoxicity of MSNs with the size of 109 nm. The results demonstrated that the particles caused a decrease in the cell viability of human hepatic L02 cells in a concentration-dependent manner, in which the cell viability, at the concentration of 120 µg/mL, compared to 5 µg/mL, decreased by ~33%. The in vivo results also demonstrated that MSNs caused toxicity in a dose-dependent manner, in which intravenous administration of MSNs, at a dose of 50 mg/kg, compared to intravenous administration of MSNs at a dose of 12.5 mg/kg, increased the serum concentration of alanine aminotransferase (ALT) and aspartate aminotransferase (AST) as markers of liver function by ~74 and ~61%, respectively, in Balb/c mice. These results indicated the hepatocellular toxicity of MSNs in both in vitro and in vivo [162,163]. Bhavsar et al. [164] synthesized MCM-41 nanoparticles of 80–120 nm and evaluated their toxicity against human hepatocellular carcinoma HepG2 cells. The results demonstrated that the nanoparticles, at the concentration of 200 µg/mL, caused cell viability of ~93%, indicating that they were safe and biocompatible. Additionally, the in vivo results demonstrated that the intravenous administration of the nanoparticles, at the dose of 40 mg/kg, in female CD-1 mice was safe and nontoxic and did not cause an increase in the serum concentration of ALT and AST [164]. Mahmoud et al. [165], in another study, explored the liver toxicity mechanisms of MSNs in rats. The animals were administered with the dose of 25, 50, 100, and 200 mg/kg MSNs for 30 days. The results demonstrated that the nanoparticles caused an increase in the serum concentration of ALT and AST in a concentration-dependent manner, in which ALT and AST in the group of animals receiving 200 mg/kg, compared to those receiving 25 mg/kg, increased by 39 and ~15%, respectively. The histopathological findings confirmed these results, in which the pathological lesions were more severe in the group of animals receiving 200 mg/kg of MSNs, compared to those receiving 25 mg/kg of MSNs. Additionally, the results of ROS, MDA, and NO measurements demonstrated that, by increasing the nanoparticle concentration, these parameters also increased, indicating that the nanoparticles increased the ROS production and oxidative stress in the liver. In contrast, the concentration of GSH, SOD, and CAT was inversely associated with the nanoparticle concentration, indicating that MSNs suppressed the antioxidants, i.e., CAT, SOD, and GSH, in the liver. The results also demonstrated that MSNs suppressed the PPARγ signaling pathway in the liver. PPARγ is able to regulate the expression of antioxidant enzymes and inhibits inflammation and fibrosis [165]. MSNs could increase the protein expression levels of TLR4, MYD88, and NF-κB p65 in the liver of rats. The activation of the TLR4/MyD88/NF-κB signaling pathway can contribute to liver fibrosis [166]. According to these results, MSNs can cause liver toxicity in a dose-dependent manner.

#### 4.3.2. Gastrointestinal Tract

The gastrointestinal tract epithelium is a barrier that allows the essential nutrients and electrolytes to selectively enter the circulation [147]. The orally administered silica nanoparticles are able to cross the gastrointestinal tract, enter the bloodstream and deposit in the liver and spleen [167]. Deng et al. [168] evaluated the subacute toxicity of MSNs in Balb/C mice and demonstrated that the continuous intragastric administration of MSNs with the size of 70 nm and the dose of 200 mg/kg/d for 2 weeks caused an increase in the serum concentrations of ALT (by ~12%), AST (by ~21%), and TNF-α (by ~65%) along with a considerable infiltration of intestinal inflammatory cells. Additionally, histopathological studies demonstrated that the nanoparticles caused intestinal damage, including inhibition in the expressions of LC3-II and Beclin1 autophagy proteins and influencing various metabolic pathways, such as pyrimidine and purine metabolism [168]. However, Cabellos et al. [169] evaluated the toxicity of MSNs with the size of 200 nm. The nanoparticles were administered orally for 5 consecutive days, and according to the results of gross pathology, genotoxicity, small intestine histopathology, and intestinal inflammation, no obvious toxicity was observed, indicating that some MSNs are safe in food and drug delivery [169]. In contrast, Yu et al. [170] demonstrated that the oral administration of MCM-41 nanoparticles with the size of 209.2 nm could disturb the gut microbiota and cause pro-inflammatory effects in rats [170]. According to these results, MSNs could cause gastrointestinal tract toxicity in a dose- and type-dependent manner and through various mechanisms, such as disturbing the expression of autophagy proteins, various metabolic pathways, and gut microbiota.

### 4.4. Toxicity to the Circulatory System

The circulatory system consists of the epidermis, blood vessels, and lymphatic and cardiovascular systems. Regardless of the administration route, silica nanoparticles, after entering the human body, are inevitably entered into the circulatory system and distributed to various organs. Various types of human endothelial cells are used to assess the toxicity of silica nanoparticles [147]. Kim et al. [171] synthesized unmodified fluorescein incorporated MSNs (FMS) and PEG/trimethyl silane-(TMS)-modified FMS (FMS@PEG/TMS) nanoparticles with the size of ~50 nm and studied their toxicity to human endothelial cells under static and microfluidic flow conditions. The results demonstrated that the FMS nanoparticles under shear stress conditions, compared to the static conditions, were more toxic towards endothelial cells by 27% after 2 h incubation. Additionally, the results demonstrated that masking the surface silanol groups of MSNs is an appropriate strategy to decrease the toxicity of nanoparticles, in which the cell viability for FMS was measured and found to be 70%, while this value for FMS@PEG/TMS under the same condition was 97%. Orlando et al. [172] demonstrated that the size effects of MSNs on the cell viability of human umbilical vein endothelial cells (HUVECs). The results demonstrated that both 250 nm and 30 nm MSNs caused cell toxicity in a concentration-dependent manner. Additionally, the results demonstrated that MSNs, with the size of 250 nm, compared to those with the size of 30 nm, caused higher toxicity by ~49%. The higher toxicity was owing to induced cell autophagy (in particular mitophagy), which can be due to higher cellular uptake (˃20%) of the nanoparticles, compared to 30 nm MSNs, with the cellular uptake of <1%. Finally, it can be concluded that the dynamic nature of the environment, the concentration, surface chemistry, and size of MSNs are critical factors, which determine the toxicity of MSNs towards the circulatory system.

## 5. Conclusions and Future Perspectives

In this review, the biomedical applications of MSNs as a drug carrier for cancer treatment have been reviewed. In particular, the application of MSNs for the delivery of some anthelmintics as repurposed drugs for cancer treatment has been discussed. MSNs are promising carriers for drug delivery and cancer therapy owing to their suitable features, such as biodegradability, biodistributability, ability to inhibit premature drug release, ability to control the drug release, adjustable size and pore volume, and high drug loading efficiency. In addition, MSNs can transform the crystalline structure of a drug to an amorphous state; thus, they could improve the solubility of hydrophobic drugs. Some of the anthelmintics as nematocidal drugs showed promising results as repurposed drugs for cancer therapy. However, their application is limited due to their low water solubility and, as a result, low bioavailability. Thus, the use of MSNs as the carrier of these anthelmintics seems to be promising as MSNs can improve the solubility of hydrophobic drugs. The results of studies [53,138,145] have shown that MSNs are promising carriers for anthelmintics as they could improve the stability, release profile, and anticancer effects. Various strategies, such as MSNs modification with PEG and BLG, have been successfully used to further improve the efficacy of these nanocarriers for the delivery of anthelmintics to treat cancers. These strategies are based on the chemical modification of these carriers. These nanoparticles can be modified with functional groups and gatekeeper molecules to control the drug release and inhibit premature drug release. However, more efficient MSN-based drug delivery systems (DDSs) are needed owing to the high prevalence and complex physiology of cancer. For this purpose, the therapeutic applications of these DDSs can be further improved through the development of multifunctional DDSs (e.g., thermosensitive, pH-responsive nanoparticles), which could specifically deliver the cargoes to cancer cells, resulting in an improvement in their efficacy. MSNs can be perfectly decomposed over one month in various environments such as simulated body fluid, cells, and body. However, the degradation of silica under physiological conditions is a relatively difficult process, and for this reason, the USFDA considers this material as GRAS. Thus, the evaluation of the safety of MSNs needs further studies to confirm the non-toxicity of MSNs. Furthermore, many advances have been made to develop sophisticated MSN-based DDSs; however, there is a severe shortage of clinical trials for these MSN-based DDSs for cancer treatment and imaging. Thus, it would be highly advantageous to focus on designing not necessarily complex MSN-based nanoformulations but, instead, focus on constructing MSN-based DDSs with increased efficacy for clinical trials. Outstanding loading capacity is one of the main benefits of MSNs compared to other nanoparticles. Additionally, gating the cargo-loaded pores to decrease the premature release is a promising property, which makes these nanoparticles ideal for targeted and localized delivery of various therapeutic molecules (e.g., imaging or contrast agents) for the treatment of different diseases [173]. The first silica-based nanoplatform, which underwent clinical trials, was a core–shell silica particle with a size of 7 nm. While the core contains Cy5 dye, the surface is modified with radiolabeled RGD peptide and PEG conjugate (124I-cRGDY-PEG). The particle demonstrated the long-term efficacy for positron emission tomography (PET) imaging of cancer cells. Moreover, the small size of the particles makes them appropriate for effective removal through the kidney; thus, the toxicity is inhibited owing to the long-term accumulation of silica nanoparticles [174]. Additionally, a gold-silica nanoparticle has been reported [175] to undergo clinical trials for the treatment of atherosclerosis using plasmonic photothermal therapy (PPTT). The nanoparticle could cause a considerable decrease in coronary atherosclerosis. However, the study needs long-term investigations. The results of the clinical trial also demonstrated that the nanoparticle was significantly potent for PPTT in the treatment of atherosclerosis and cancer [176,177]. Although MSNs demonstrated tremendous potential in the lab-scale and in vivo studies as controlled drug delivery systems, industrial translation encounters considerable challenges, and the most important challenge is the scaling up of the synthesis process. The lab-optimized synthesis methods, when used for scaling up to industrial levels, encounter remarkable variations in the nanoparticles’ size distribution and batch-to-batch variability. As the synthesis of MSNs is a long and multi-step process, the optimization of the synthesis is a very tedious and expensive process, compared to other polymeric nanoparticles. Moreover, the probable long-term toxicity, owing to MSN accumulation within the body, is a reason for concern. However, recent clinical trials for MSNs, with a size of below 10 nm, certainly show a way beyond. The results of these clinical trials can further help to minimize the treatment cost by decreasing the drug dose.

## Figures and Tables

**Table 1:** Name, chemical structure, class, and mode of action of various anthelmintics.

Name	Chemical Structure	Class	Mode of Action
Albendazole	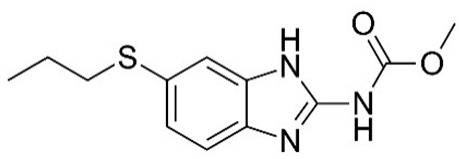	Benzimidazoles	Impairment of the polymerization of β-tubulin and α-tubulin [122].
Thiabendazole	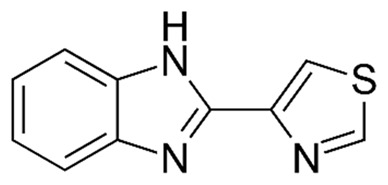	Benzimidazoles	Most likely influencing the helminth-specific mitochondrial enzyme fumarate reductase and by which inhibiting the citric acid cycle and mitochondrial respiration, resulting in helminth’s death [123].
Fenbendazole	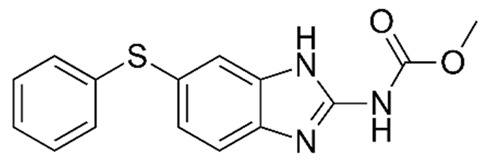	Benzimidazoles	Binding to nematode β-tubulin and preventing microtubule formation [124].
Niclosamide	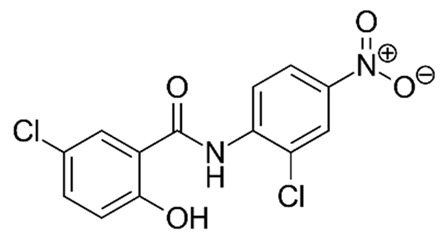	Salicylanilides	Inhibiting the glucose uptake; therefore, uncoupling energy-generating oxidative phosphorylation in intestinal worms; thus, making the worms hungry for ATP [125].
Ivermectin	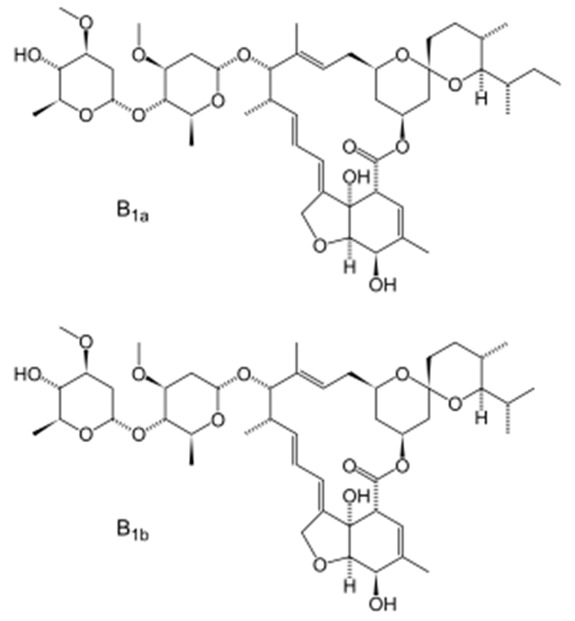	Macrocyclic lactones	Binding and activating chloride ion channels in nematodes [126].
Abamectin	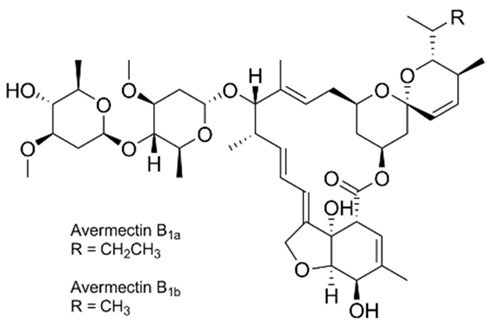	Macrocyclic lactones	Activating glutamate-gated chloride channels and modulating other Cys-loop ion channels [127].

## Data Availability

Not applicable.
